# Diversity-Aware Marine Predators Algorithm for Task Scheduling in Cloud Computing

**DOI:** 10.3390/e25020285

**Published:** 2023-02-02

**Authors:** Dujing Chen, Yanyan Zhang

**Affiliations:** School of Electronics and Information Engineering, Nanjing University of Information Science & Technology, Nanjing 210044, China

**Keywords:** cloud computing, task scheduling, marine predators algorithm

## Abstract

With the increase in cloud users and internet of things (IoT) applications, advanced task scheduling (TS) methods are required to reasonably schedule tasks in cloud computing. This study proposes a diversity-aware marine predators algorithm (DAMPA) for solving TS in cloud computing. In DAMPA, to enhance the premature convergence avoidance ability, the predator crowding degree ranking and comprehensive learning strategies were adopted in the second stage to maintain the population diversity and thereby inhibit premature convergence. Additionally, a stage-independent control of the stepsize-scaling strategy that uses different control parameters in three stages was designed to balance the exploration and exploitation abilities. Two case experiments were conducted to evaluate the proposed algorithm. Compared with the latest algorithm, in the first case, DAMPA reduced the makespan and energy consumption by 21.06% and 23.47% at most, respectively. In the second case, the makespan and energy consumption are reduced by 34.35% and 38.60% on average, respectively. Meanwhile, the algorithm achieved greater throughput in both cases.

## 1. Introduction

Cloud computing is a large resource pool that is dynamic and scalable, and the data center of a third-party service operator provides resources. Users can directly use the computing and storage resources of the cloud servers through the network [1], and internet of things (IoT) applications are deployed on the cloud. It has facilitated applications based on artificial intelligence and IoT [2], whereas, with the many conveniences brought by cloud computing, the increasing task to be processed and expansion of cloud resources also make scheduling cloud resources challenging.

Cloud resource scheduling has two main layers. The first layer schedules appropriate virtual resources for tasks submitted by users, and the second layer schedules appropriate hosts for virtual resources. This study focuses on the first layer because task scheduling in cloud computing (TSCC) directly affects the quality of services (QoS) parameters [3], such as the makespan, energy consumption, resource utilization rate, task response time, and task rejection rate. Under the constraints of QoS parameters, mapping a set of tasks to a suitable virtual resource is an NP-hard problem [4,5], and the algorithm suffers from dimensionality breakdown as the problem sizes increase.

Hence, ideas that apply meta-heuristic algorithms (MHAs) to TS have emerged to efficiently allocate available resources to complex and diverse incoming tasks within a reasonable time and with limited resources because of some of its inherent properties, such as its stochastic behavior. In addition, it has no dependency on the problem being solved and can search the solution space quickly to find the approximate optimal solution. Classical meta-heuristics, such as GA [6], PSO [7], novel whale optimization algorithm (WOA) [8], and Harris hawks optimization (HHO) [9] have been applied to TSCC successfully.

Although many MHA have been applied to cloud computing task scheduling problems, these algorithms tend to fall into the local optimum, resulting in high energy consumption of the system, long task completion time and other problems, affecting the overall optimization effect. This study applies a novel MHA, the marine predators algorithm (MPA), to solve TS in cloud computing. The motivation for this is that the MPA algorithm is capable of avoiding falling into the local optimum and achieves excellent performance in the optimization of complex problems [10]. To further strengthen the performance of the MPA algorithm and find a better solution that satisfies the QoS parameters, we propose a diversity-aware marine predators algorithm (DAMPA) to reduce makespan and energy consumption and increase throughput. Two strategies were used in the DAMPA to raise the variety of the population and prevent premature convergence. First, the predator crowding degree ranking strategy was designed to determine whether a predator performs exploration or exploitation in the second stage. Second, a comprehensive learning strategy was applied to enable predators to share the best experience. In addition, a stage-independent control of the stepsize-scaling strategy in DAMPA, which uses different control parameters in three stages, was designed to balance the exploration and exploitation abilities. The main contributions of this study are described below:

(1) The DAMPA is proposed for resolving TSCC in reducing makespan and energy consumption and increasing throughput.
(2) To avoid premature convergence, the predator crowding degree ranking strategy is designed, and the comprehensive learning strategy is applied.
(3) A stage-independent control of the stepsize-scaling strategy is designed to balance the exploration and exploitation abilities.

This article’s structure is as follows: Section 2 discusses the similar works on TSCC. Section 3 introduces the problem formulation of the TS. Section 4 first introduces the MPA algorithm and then elaborates on our proposed DAMPA algorithm. Section 5 presents an experimental evaluation consisting of tests for multiple indicators. Section 6 is the conclusion of this work.

## 2. Related Works

TSCC has received considerable attention from scholars. Marahatta et al. [11] proposed a scheduling approach that first classifies heterogeneous tasks and virtual machines (VMs), then similar types of tasks are combined and scheduled. Additionally, it exploits the energy efficiency and optimal operating frequency of heterogeneous physical hosts to save energy when creating and deleting VMs. Hussain et al. [12] divided TS into two stages according to the optimization objectives. The first stage of scheduling focuses on reducing the execution time of the tasks, and the second stage is to reduce energy consumption.

In addition to classification-based and phased scheduling methods, meta-heuristic algorithms are key in solving TS problems in cloud computing because of their superior performance in the optimization of complex problems. For example, Chen et al. [13] used the WOA algorithm for improving the efficiency of task execution. Abdullah et al. [14] combined the PSO algorithm with the Pareto optimal frontier, and a mutation operator was introduced to avoid premature convergence. Laili et al. [15] designed a parallel TS algorithm, which divides tasks into several groups, finds the best solution with meta-heuristic algorithms for each group and then merges all sub-solutions into the final solution. This algorithm decreases the total execution time and energy consumption, but the TS model is complex. Ali et al. [16] proposed an optimization model based on NSGA-II that realizes automatic mapping between tasks and cloud nodes and minimizes the total execution time.

To reduce the makespan, Xiong et al. [17] introduced Johnson’s rule to GA, which adds a new crossover and the mutation operation. Pirozmand et al. [18] first prioritized tasks and then assigned tasks to VMs using GA. Pang et al. [19] introduced the estimated distribution algorithm (EDA) to the GA algorithm to initialize the solution. Xu et al. [20] prioritized the TS sequence according to the priority algorithm and used the ant colony algorithm (ACO) to obtain a global optimal scheduling scheme under certain constraints.

Because of the limitations of a single meta-heuristic algorithm, several scholars have adopted a hybrid bio-inspired algorithm. For example, Attiya et al. [21] combined the manta-ray foraging optimizer (MRFO) and Salp Swarm algorithm (SSA), which strengthens the exploitation capability, and the results indicate that the approach outperforms the existing algorithms. Similarly, Walia et al. [22] combined GA with the flower pollination algorithm (FPA), and the crossover and mutation of the GA are introduced in the FPA. Domanal et al. [23] used an improved PSO to find a better solution for mapping tasks to VMs and adopted a modified hybrid bio-inspired algorithm for resource allocation according to the needs of the tasks. Fu et al. [24] combined the PSO and GA algorithms to reduce the task completion time. Although hybrid algorithms have achieved better results in several indicators, they are also overly complex.

Based on the above analysis, various algorithms have been applied to the TS. The optimization of makespan, energy consumption, and throughput, three important indicators of TS, is still insufficient. Therefore, a new method based on MPA was proposed for TSCC.

## 3. Problem Formulation

### 3.1. Task Scheduling

In the cloud, the data center receives the tasks uploaded through the network, and the broker allocates the tasks to the appropriate available resources according to the task requirements and information of the VMs and optimization goals. TS in the cloud can be summarized as the mapping of a group of tasks $\left\{T_{1},T_{2},T_{3}\ldots ,T_{m}\right\}$ to $\left\{VM_{1},VM_{2},VM_{3}\ldots ,VM_{n}\right\}$, *m* is the total number of tasks to be processed, *n* is the total number of virtual machines. $T_{i}$ has a corresponding task size, and the key factors of $VM_{j}$ are processing speed, RAM, and CPU processing elements. One task can only be assigned to one VM, and each VM executes multiple tasks. In this study, our goal is to map tasks to VMs to decrease the makespan and energy consumption and increase throughput.

### 3.2. Mathematical Model

In the TS process, $D_{ij}$ represents the decision variable of the allocation process, which is expressed as follows:

(1) $$D_{ij}=\left\{\begin{array}{cc} 1, & T_{i}\;\mathrm{to}\;VM_{j}\\ 0, & \mathrm{otherwise} \end{array}\right.$$

The ranges of the subscripts *i* and *j* of the decision variable $D_{ij}$ are the number of tasks and the number of virtual machines respectively. When the number of virtual machines or tasks increases, more decision values are required. Since each task must be handled by just one VM, and since the schedule allocates all the tasks, we have

(2) $$\sum_{j=1}^{n}D_{ij}=1$$

(3) $$\sum_{i=1}^{m}\sum_{j=1}^{n}D_{ij}=m$$

The processing time of $T_{i}$ on $VM_{j}$ can be calculated by the following:

(4) $$t_{ij}=\frac{W_{i}}{S_{j}}$$

where $t_{ij}$ represents the processing time of $T_{i}$ on $VM_{j}$, $W_{i}$ represents the task size of task $T_{i}$, $S_{j}$ represents the processing speed of $VM_{j}$.

#### 3.2.1. Makespan

We assumed that each VM starts executing the first task from time 0 until the last task is executed, and the time spent during this period is the total execution time of $VM_{j}$. The makespan can be calculated as follows:

(5) $$t_{j}=\sum_{i=1}^{m}t_{ij}\cdot D_{ij}$$

(6) $$t_{M}=\max\left(t_{j}\right),j=1,2\ldots n.$$

where $t_{j}$ represents the total execution time of $VM_{j}$, $t_{M}$ represents the makespan.

#### 3.2.2. Energy Consumption

The energy consumed by VMs includes the idle state and the working state. The consumption of VMs in the idle state is approximately 0.6 times the working state [25]. The total energy consumption (TEC) is calculated as follows:

(7) $$TE_{j}=\left(t_{j}\cdot \alpha +\left(t_{M}-t_{j}\right)\cdot \beta \right)\cdot S_{j}$$

(8) $$TEC=\sum_{j=1}^{n}TE_{j}$$

where $TE_{j}$ is the energy consumption of the $VM_{j}$, $\alpha =10^{-8}\cdot S_{j}^{2}$, β=0.6·α.

#### 3.2.3. Throughput

Throughput metric measures, the performance of the cloud system to complete tasks over a period is reflected in work completed by the cloud data center per unit of time. It can be calculated as follows:

(9) $$Throughput=\frac{Task\;quantity}{t_{M}}$$

#### 3.2.4. Fitness Function

The fitness function measures the performance of individuals. Based on our optimization goals of energy consumption, makespan, and throughput, as makespan is a key factor in throughput, we set the objective function to the following to evaluate the candidate solutions:  

(10) $$Fitness=\lambda_{1}\cdot TEC+\lambda_{2}\cdot t_{M}$$

where $\lambda_{1}+\lambda_{2}=1$, we set $\lambda_{1}$ = 0.5 and $\lambda_{2}$ = 0.5.

## 4. The Proposed Algorithm

This part first introduces the MPA algorithm to present its optimization process, and then the proposed DAMPA algorithm is elaborated. There are three differences between DAMPA and MPA: the predator crowding degree ranking strategy, comprehensive learning strategy, and stage-independent control of the stepsize-scaling strategy.

### 4.1. The Overview of MPA

#### 4.1.1. Elite and Prey Matrix

Based on the theory of survival of the fittest, the best predators are better at foraging in natural survival. Therefore, the optimal solution is set as the top predator. The optimal solution for each predator constructs the El matrix. The El matrix is as follows:

(11) $$El=\left[\begin{array}{cccc} I_{1,1}^{E} & I_{1,2}^{E} & \cdots \; & I_{1,d}^{E}\\ I_{2,1}^{E} & I_{2,2}^{E} & \cdots \; & I_{2,d}^{E}\\ \vdots & \vdots & \ddots \\ I_{k,1}^{E} & I_{k,2}^{E} & \cdots \; & I_{k,d}^{E} \end{array}\right]$$

where $\vec{I_{i}^{E}}=\left[I_{i,1}^{E},I_{i,2}^{E}\ldots I_{i,d}^{E}\right]$, *k* marks the number of predators in the population, and dimension *d* represents the number of variables in the solution $\vec{I_{i}^{E}}$.

The prey matrix ($P_{e}$) and the El matrix are similar, the Pe matrix saves the new value produced each iteration, and the El matrix represents the historical optimal solution of the predators. The $P_{e}$ matrix is expressed as follows:

(12) $$Pe=\left[\begin{array}{cccc} I_{1,1}^{P} & I_{1,2}^{P} & \cdots \; & I_{1,d}^{P}\\ I_{2,1}^{P} & I_{2,2}^{P} & \cdots \; & I_{2,d}^{P}\\ \vdots & \vdots & \ddots \\ I_{k,1}^{P} & I_{k,2}^{P} & \cdots \; & I_{k,d}^{P} \end{array}\right]$$

where $\vec{I_{j}^{P}}=\left[I_{j,1}^{P},I_{j,2}^{P}\ldots I_{j,d}^{P}\right]$. In the initialization phase of the algorithm, the values in the $P_{e}$ matrix will be randomly generated within the specified range, the optimal solution $\vec{I^{E}}$ will be obtained through calculating each $\vec{I^{P}}$, and the El matrix will be initialized by duplicating $\vec{I^{E}}$.

#### 4.1.2. Optimization Process

In the first stage, all predators perform exploration. Taking advantage of the Brownian motion step sizes helps predators approach the optimal target more quickly at the initial stage when they are far from the optimal target. Formulated as follows, when $iter<\frac{1}{3}Maxiter$,

(13) $$\begin{array}{c} \vec{L_{i}}=\vec{M_{B}}⊗\left(\vec{El_{i}}-\vec{M_{B}}⊗\vec{Pe_{i}}\right) \end{array}$$

(14) $$\begin{array}{c} \vec{Pe_{i}}=\vec{Pe_{i}}+P*\vec{R}⊗\vec{L_{i}} \end{array}$$

where $\vec{L_{i}}$ represents the step size of each predator, $\vec{M_{B}}$ is a vector composed of standard normal distribution random numbers (RND), $\vec{R}$ is a uniform RND vector in [0,1], and *P* is a constant, $\vec{Pe_{i}}$ and $\vec{El_{i}}$ are the row vector of the matrix, iter is the current number of iterations, and Maxiter is the maximum number of iterations.

The second stage involves the shift from exploration to exploitation. Half of the predators adopted the exploitation strategy, and the remainder adopted the exploration strategy. When $\frac{1}{3}Maxiter<iter<\frac{2}{3}Maxiter$, we have the following.

First one-half of predators:

(15) $$\begin{array}{c} \vec{L_{i}}=\vec{M_{L}}⊗\left(\vec{El_{i}}-\vec{M_{L}}⊗\vec{Pe_{i}}\right) \end{array}$$

(16) $$\begin{array}{c} \vec{Pe_{i}}=\vec{Pe_{i}}+P*\vec{R}⊗\vec{L_{i}} \end{array}$$

Other predators:

(17) $$\begin{array}{c} \vec{L_{i}}=\vec{M_{B}}⊗\left(\vec{M_{B}}⊗\vec{El_{i}}-\vec{Pe_{i}}\right) \end{array}$$

(18) $$\begin{array}{c} \vec{Pe_{i}}=\vec{El_{i}}+P*CF⊗\vec{L_{i}} \end{array}$$

where $\vec{M_{L}}$ is a RND vector generated based on the Levy distribution and CF controls the step sizes of the predators and can be calculated as follows:

(19) $$\begin{array}{c} CF={\left(1-\frac{iter}{Maxiter}\right)}^{2\frac{iter}{Maxiter}} \end{array}$$

In the third stage, all individuals are in a state of Levy movement and execute the exploitation strategy, when $iter>\frac{2}{3}Maxiter$:

(20) $$\begin{array}{cc} \vec{L_{i}} & =\vec{M_{L}}⊗\left(\vec{M_{L}}⊗\vec{El_{i}}-\vec{Pe_{i}}\right) \end{array}$$

(21) $$\begin{array}{cc} \vec{Pe_{i}} & =\vec{El_{i}}+P*CF⊗\vec{L_{i}} \end{array}$$

In simulating the survival of predators, the eddy taking shape or fish aggregation device (FADs) effect in the environment also causes predators to adjust to their movement states. A broad range of jumps helps the algorithm avoid stagnancy in the local optima. The FADs effect is expressed as follows:

(22) $$\begin{array}{c} \vec{Pe_{i}}=\left\{\begin{array}{cc} \vec{Pe_{i}}+CF\left[\vec{Pe_{l}}+\vec{R}⊗\left(\vec{Pe_{u}}-\vec{Pe_{l}}\right)\right]⊗\vec{U}, & \mathrm{if}\;r\leq FADs\\ \vec{Pe_{i}}+\left[FADs(1-r)+r\right]\left(\vec{Pe_{i1}}-\vec{Pe_{i2}}\right), & \mathrm{if}\;r>FADs \end{array}\right. \end{array}$$

where FADs=0.2 represents the probability that the solution is affected during the update process, *r* is a RND in [0,1], and *R* is a RND vector in [0,1]. $\vec{Pe_{l}}$ consists of the minimum value in each dimension and $\vec{Pe_{u}}$ consists of the maximum value in each dimension. *U* is a binary number, and i1 and i2 are random indices. Finally, the pseudocode of the MPA is expressed as Algorithm 1.
**Algorithm 1** MPA1:initialize the Pe, $\vec{S}$, P, FADs, Maxiter, Tf, Tp2:**while** iter<Maxiter **do**3:   Compute the fitness4:   Construct Elite matrix5:   Save memory6:   update CF using (19)7:   **if** $iter<\frac{1}{3}Maxiter$ **then**8:      Update Pe using (13) and (14)9:   **else if** $\frac{1}{3}Maxiter<iter<\frac{2}{3}Maxiter$ **then**10:     First one-half of predators:11:     Update Pe using (15) and (16)12:     other predators:13:     Update Pe using (17) and (18)14:   **else if** $iter>\frac{2}{3}Maxiter$ **then**15:     Update Pe using (20) and (21)16:   **end if**17:   Compute the fitness18:   Saving memory19:   Applying FADs effect using (22)20:   iter++21:**end while**

### 4.2. The DAMPA Algorithm

#### 4.2.1. The Predator Crowding Degree Ranking Strategy

In the optimization process of the MPA, the second stage, which carries out the transition from exploration to exploitation, is indispensable. Here, the MPA algorithm neglects the population diversity when selecting predators to perform exploitation and exploration. If predators that are closer to each other are selected for exploitation in the second stage, the population diversity will be prematurely reduced. Therefore, to raise the variety of the population and thereby avoid premature convergence, the predator crowding degree ranking strategy is adopted in the DAMPA. The crowding degree of a predator describes the magnitude of the location difference between the predator and the remaining predators in the population, if a predator is close to other predators, it has a larger crowding degree. After the end of the first stage in the DAMPA, the crowding degree of each predator is calculated using (23) and (24), and rank the predators according to their degree of crowding from small to large. The predators with lower rankings execute the exploration, and the remaining predators execute the exploitation. The number of predators executing the two strategies was the same. Algorithm 2 shows the crowding degree ranking algorithm. We assume that $\vec{Pe_{i}}$ is the current predator and $\vec{Pe_{j}}$ is the other predator. The mathematical expression for crowding degree (CD) is as follows:

(23) $${DE}_{\vec{Pe_{i}}}=\sum_{j=1}^{m}{\left(\sum_{k=1}^{d}{\left(I_{i,k}-I_{j,k}\right)}^{2}\right)}^{0.5}\left(i\neq j\right)$$

(24) $$CD_{\vec{Pe_{i}}}=\frac{1}{PopulationSize-1}*{DE}_{\vec{Pe_{i}}}$$

where $\vec{Pe_{i}}=\left[I_{i,1}^{P},I_{i,2}^{P}\ldots I_{i,d}^{P}\right]$,$\vec{Pe_{j}}=\left[I_{j,1}^{P},I_{j,2}^{P}\ldots I_{j,d}^{P}\right]$.
**Algorithm 2** Crowding degree ranking algorithm.1:initialize the CD, Ranking (two arrays of length PopulationSize)2:**for** i = 0 to PopulationSize **do**3:   Computing $CD_{i}$ based on (23) and (24)4:**end for**5:**for** j=0 to PopulationSize **do**6:   **for** k=0 to PopulationSize **do**7:     **if** $CD_{j}<CD_{k}$ **then**8:        $Ranking_{j}++$9:     **end if**10:  **end for**11:**end for**12:**return** Ranking

#### 4.2.2. Comprehensive Learning Strategy

In DAMPA, a comprehensive learning strategy is also applied in the second stage, which enables the exchange of best experiences between predators. This maintains the population diversity and avoids premature convergence [26]. Predators with high crowding degrees are given a larger learning rate to maximize the population diversity, and the learning rate of predators can be calculated by the following:

(25) $$\begin{array}{c} Pe_{l_{i}}=c+e*\frac{exp(\frac{10(Ranking_{i}-1)}{PopulationSize-1})}{exp(10)-1} \end{array}$$

where c=0.05, and e=0.5.

A comprehensive learning strategy is shown in Algorithm 3. First, the learning rate $Pe_{l}$ of the predators was calculated. Subsequently, a random value *r* is generated, and predators $Pe_{k1}$ and $Pe_{k2}$ are randomly selected. When $r<Pe_{l}$, the fitness value of each predator was calculated. If the fitness value of $Pe_{k1}$ is less than $Pe_{k2}$, then $Pe_{k1}$ is used as an exemplar. Otherwise, $Pe_{k2}$ was used as an example. If $r>Pe_{l}$, then the predator is used as an exemplar.

Next, in the second stage, we applied comprehensive learning, in which (15) and (17) are replaced by (26) and (28), respectively.

First one-half of predators:

(26) $$\begin{array}{cc} \vec{L_{i}} & =\vec{M_{L}}⊗\left(\vec{El_{i}}-\vec{M_{L}}⊗\vec{Pe_{i}}+c1*R⊗\left(\vec{Pe_{bFi}}-\vec{Pe_{i}}\right)\right) \end{array}$$

(27) $$\begin{array}{c} c1=\frac{1}{1+e^{-\theta *\frac{iter}{Maxiter}}}+2*{\left(\frac{iter}{Maxiter}-1\right)}^{2} \end{array}$$

where $\vec{Pe_{bFi}}=exemplar$, θ=0.0001.

Other predators:

(28) $$\begin{array}{cc} \vec{L_{i}} & =\vec{M_{B}}⊗\left(\vec{M_{B}}⊗\vec{El_{i}}-\vec{Pe_{i}}+c2*R⊗\left(\vec{Pe_{bFi}}-\vec{Pe_{i}}\right)\right) \end{array}$$

(29) $$\begin{array}{c} c2=\frac{1}{1+e^{-\theta *\frac{iter}{Maxiter}}}+2*{\left(\frac{iter}{Maxiter}\right)}^{2} \end{array}$$

**Algorithm 3** Comprehensive learning strategy.1:**for** i=0 to PopulationSize **do**2:   Calculate $\vec{Pe_{l_{i}}}$ using (25)3:   Select two random agents with indexs k1,k24:   **if** $\vec{P_{l_{i}}}>r$ **then**5:     Calculate the fitness of $\vec{Pe_{k1}}$ and $\vec{Pe_{k2}}$6:     **if** $fitness_{Pe_{k1}}<fitness_{Pe_{k2}}$ **then**7:        $exemplar=\vec{Pe_{k1}}$8:     **else**9:        $exemplar=\vec{Pe_{k2}}$10:     **end if**11:   **end if**12:   **if** $\vec{P_{l_{i}}}<r$ **then**13:     $exemplar=\vec{Pe_{i}}$14:   **end if**15:**end for**

#### 4.2.3. Stage-Independent Control of Stepsize-Scaling Strategy

**Figure 1:**
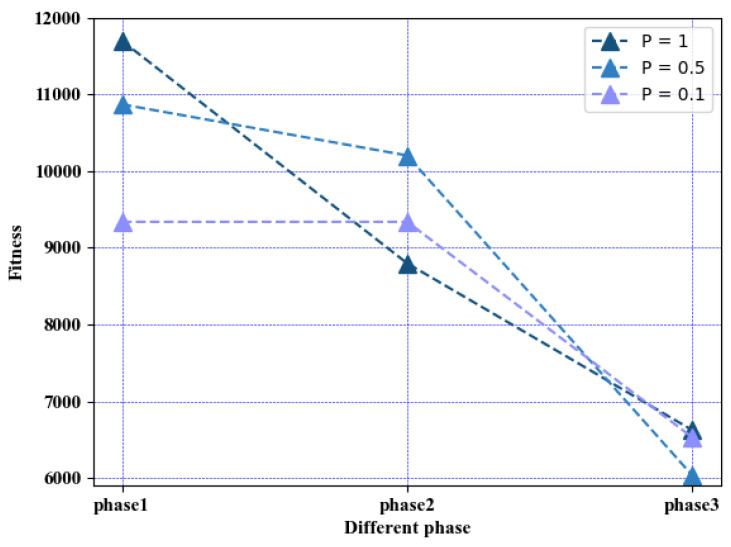
Phase convergence performance of different step sizes scaling control parameters.

In MPA, *P* is the parameter of step size scaling, which controls the enlargement or reduction of the step sizes in three stages simultaneously. Figure 1 shows the convergence performance at each stage with different step sizes scaling control parameters. When P=0.1, the algorithm performs better in the first stage and has a strong exploration ability, but the exploitation ability is insufficient in the third stage. When P=0.5, the algorithm has a strong exploitation ability, but the exploration ability in the first stage is insufficient. Therefore, to balance the exploration and exploitation capabilities of the MPA algorithm, the stage-independent control of the stepsize-scaling strategy is designed in DAMPA to optimize the performance at each stage by using $P_{1}$, $P_{2}$, and $P_{3}$ to control the scaling of the step sizes of the three stages. The control parameter is set to $P_{1}$ in the first stage. The second stage corresponds to $P_{2}$. In the third stage, we have $P_{3}$.

Based on the above operations, we use (30), (31), (32), and (33) to replace (14), (16), (18), and (21), respectively. The complete DAMPA is presented in Algorithm 4.

(30) $$\begin{array}{c} \vec{Pe_{i}}=\vec{Pe_{i}}+P1*\vec{R}⊗\vec{L_{i}} \end{array}$$

(31) $$\begin{array}{cc} \vec{Pe_{i}} & =\vec{Pe_{i}}+P2*\vec{R}⊗\vec{L_{i}} \end{array}$$

(32) $$\begin{array}{cc} \vec{Pe_{i}} & =\vec{El_{i}}+P2*CF⊗\vec{L_{i}} \end{array}$$

(33) $$\begin{array}{cc} \vec{Pe_{i}} & =\vec{El_{i}}+P3*CF⊗\vec{L_{i}} \end{array}$$

**Algorithm 4** DAMPA
1:Initialize the Pe,$\vec{S}$, P1, P2, P3, FADs, Maxiter, Tfs, Tp2:**while** iter<Maxiter **do**3:   Compute the fitness of $\vec{Pe}$4:   Construct El matrix5:   Save memory6:   update CF using (19)7:   **if** $iter<\frac{1}{3}Maxiter$ **then**8:     Update Py using (13) and (30)9:   **else if** $iter=\frac{1}{3}Maxiter$ **then**10:     Calculate the crowding degree ranking11:  **else if** $\frac{1}{3}Maxiter<iter<\frac{2}{3}Maxiter$ **then**12:     **if** $Ranking_{\vec{Pe_{i}}}<\frac{1}{2}PopulationSize$ **then**13:        Update Py using (26) and (31)14:     **else**15:        Update Py using (28) and (32)16:     **end if**17:  **else if** $iter>\frac{2}{3}Maxiter$ **then**18:     Update Py using (20) and (33)19:  **end if**20:   Compute $\vec{Pe}$21:   Save memory22:   Apply FADs effect using (22)23:   iter++24:
**end while**



#### 4.2.4. Complexity Analysis

The complexity of the MPA algorithm is O(T(Pd×Ps+C×Ps)), the crowding degree ranking algorithm is O(Ps×Ps×Pd), and the comprehensive learning strategy is $O(\frac{1}{3}\times T\times Ps)$. The DAMPA complexity is O(T(Pd×Ps+C×Ps)+Ps×Ps×Pd), where *T* is the maximum iterations, Ps is the amount of predators, *C* is the evaluated cost, and Pd is the dimension of predators.

## 5. Experiment and Analysis

This section introduces the dataset settings, parameter settings, and experimental results compared with those of other algorithms. The proposed DAMPA is written based on the Java language, and the experimental computer specifications are inter-core i7-9700CPU@3.0GHZ, 32 GB RAM, Windows 10 64-bit operating system, Cloudsim4.0.

### 5.1. Data Set

The establishment of the dataset was mainly from the perspectives of tasks and VMs. It was considered in two cases to simulate resource-limited and resource-rich situations.

#### 5.1.1. Case1

The number of VMs is fixed at 50, and the processing speed of each VM to 2000+j×40, where *j* is the index of the VMs and j=1,2,3…50. The tasks is set to 100, 200, 300, 400, 500, 600, 700, and 800. The size of the tasks was randomly generated within [200, 12,000].

#### 5.1.2. Case2

The number of tasks is fixed at 200, and the size of each task was set to 1000+i×5, where *i* is the index of the tasks and i=1,2,3…200. The VM is set to 50, 60, 70, 80, 90, 100, 110, 120, and 130. The $S_{j}$ of the VMs was generated in [200, 12,000] randomly.

### 5.2. Parameter Setting

We compared the proposed algorithm with existing algorithms including IMMPA [27], WOA, MRFOSSA [21], and HHO to verify our algorithm performance. Table 1 lists the parameter settings of the algorithms, the population size is 50, and the maximum iteration is ten thousand. Each algorithm was run twenty times independently, and the results were averaged.

### 5.3. Discretization

At this stage, since the proposed optimization algorithm contains continuous values, it needs to be discretized. The continuous values of predator need to be converted to discrete values (VMs number). First, the predator vector is normalized as follows:

(34) $${\mathrm{normalized}}_{NV}=\frac{NV-\min}{\max-\min}$$

where min and max are the minimum and maximum values in the predator vector $\vec{I}$, respectively. NV is the new value generated by each update in the vector $\vec{I}$, and ${\mathrm{normalized}}_{NV}$ represents the normalized value. After that, the following equation is used to scale the value in vector $\vec{I}$:

(35) $${\mathrm{scaled}}_{NV}=\;\mathrm{normalized}{\;}_{NV}*\left(n-1\right)+1$$

where ${\mathrm{scaled}}_{NV}$ is the scaled value, *n* is the number of virtual machines.

### 5.4. Experimental Results

**Figure 2:**
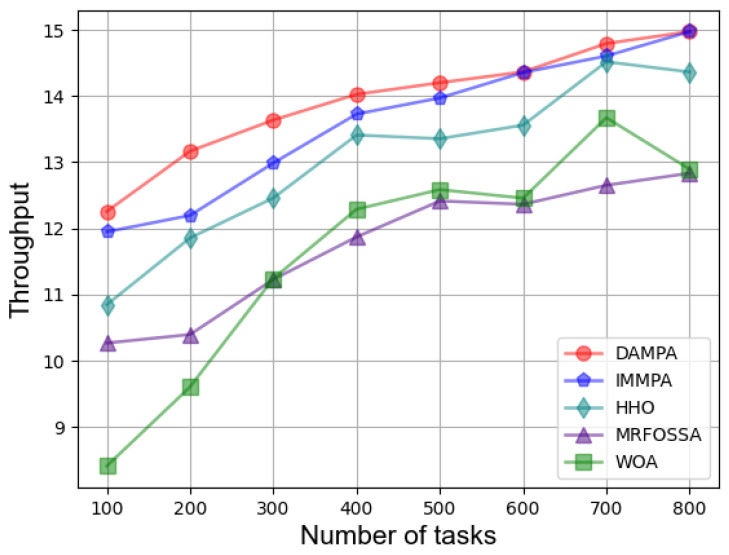
Comparison based on throughput under different number of tasks.

In the first case, the fitness under different numbers of tasks is shown in Table 2, which indicates that DAMPA obtains a lower fitness value than IMMPA, HHO, MRFOSSA, and WOA. DAMPA has a small improvement based on IMMPA, and a large improvement based on HHO, MRFOSSA, and WOA. Table 3 shows the makespan under the different number of tasks. The makespan value increases when the amount of tasks to be processed increases. DAMPA obtains the lesser makespan value. To more accurately describe the effect of optimization, Table 4 compares the percentage of makespan decrease of different algorithms, when the task size is minimal and the makespan value is reduced by 2.60%, 11.56%, 16.27%, and 31.37%, over IMMPA, HHO, MRFOSSA, and WOA. With the expansion of the scale of the scheduling problem, finding a solution becomes difficult for all algorithms, and the overall optimization effect of DAMPA is constantly weakening. DAMPA improved by 7.38% at most based on IMMPA and improved by 1.86–11.56%, 12.59–21.06%, 7.58–31.37% based on HHO, MRFOSSA, and WOA respectively. Table 5 lists the TEC under the different numbers of tasks. More energy is consumed with an increasing number of tasks. DAMPA consumes less energy than IMMPA, HHO, MRFOSSA, and WOA. More specifically, Table 6 describes the improvement percentage in TEC. When the task size is minimal, the TEC is reduced by 0.99%, 17.64%, 23.47%, and 33.14% over IMMPA, HHO, MRFOSSA, and WOA, respectively. As the scale of the problem increases, the optimization effect of DAMPA weakens on the whole. Compared with IMMPA, HHO, MRFOSSA, and WOA, the improvement ranges are 0.07–6.33%, 1.85–17.64%, 11.57–23.47%, and 5.60–33.14%, respectively. The throughput of the system under different numbers of tasks is depicted in Figure 2, which shows that DAMPA achieves a greater throughput than IMMPA, HHO, MRFOSSA, and WOA.

**Figure 3:**
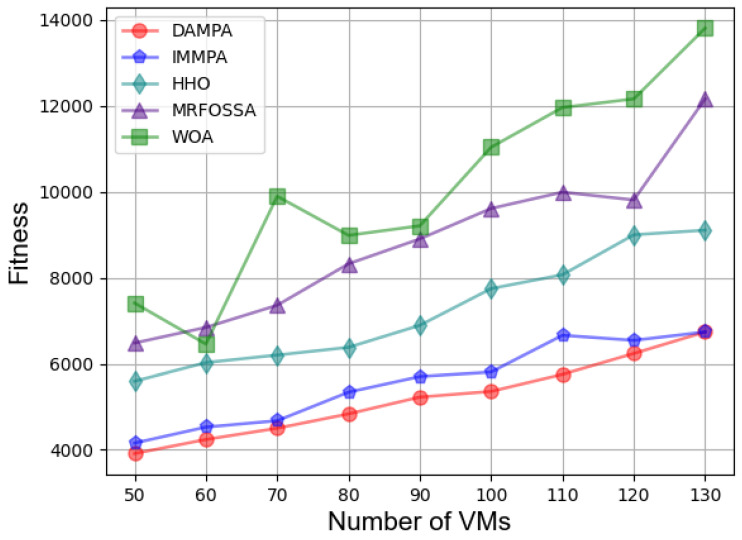
Comparison based on fitness under different numbers of VMs.

**Figure 4:**
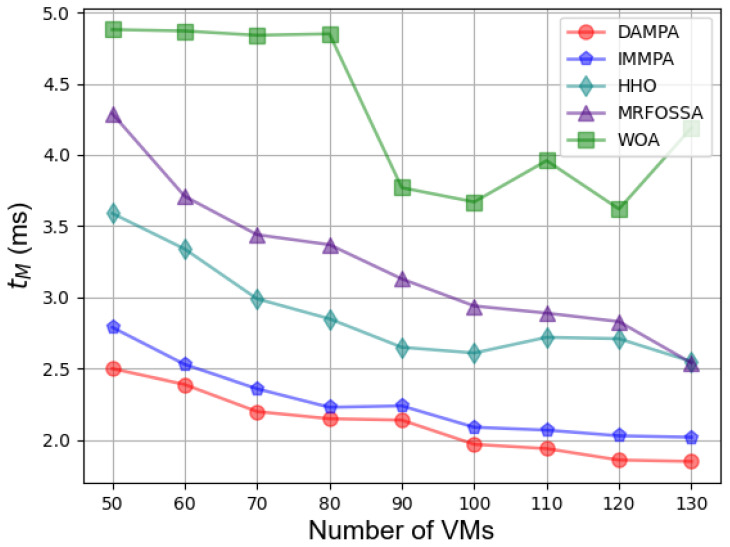
Comparison based on makespan under different numbers of VMs.

**Figure 5:**
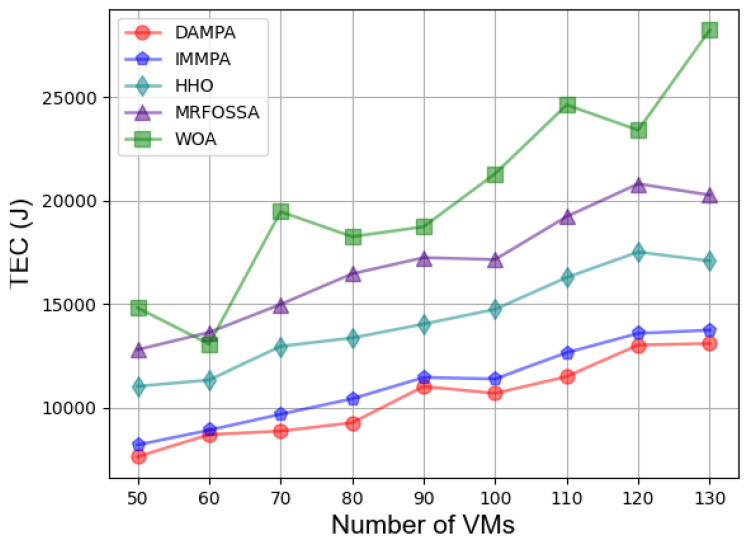
Comparison based on TEC under different numbers of VMs.

**Figure 6:**
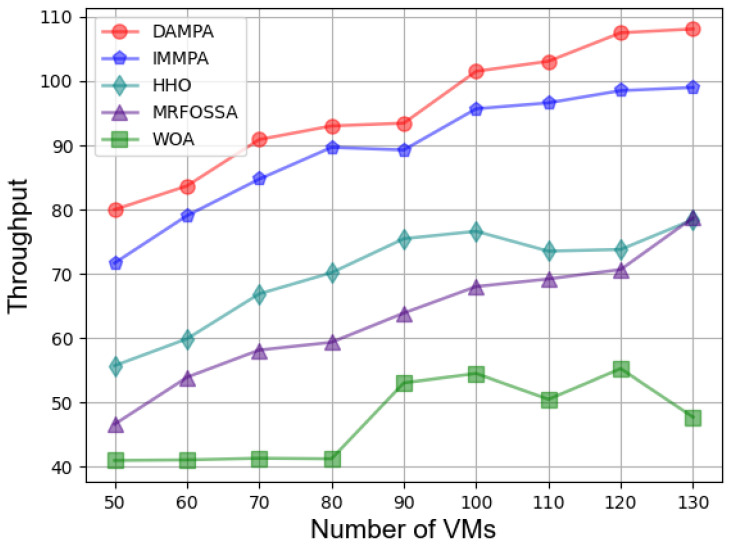
Comparison based on throughput under different numbers of VMs.

In the second case, Figure 3 shows the effect of different numbers of VMs on fitness. The fitness value keeps increasing with the number of VMs increasing, which is caused by more energy consumption. The fitness value of DAMPA is below IMMPA, HHO, MRFOSSA, and WOA. The makespan for different numbers of VMs is shown in Figure 4, which indicates that DAMPA obtains a lower makespan value than IMMPA, HHO, MRFOSSA, and WOA. More VMs enable DAMPA, IMMPA, MRFOSSA, and HHO to find better scheduling schemes, makespan continues to decrease, and the WOA algorithm has the largest fluctuation. Table 7 lists the percentage reduction of makespan, the increase in VMs scale has no obvious impact on the optimization effect of DAMPA, the makespan was reduced by 6.81%, 26.92%, 34.35%, and 50.60% on average over IMMPA, HHO, MRFOSSA, and WOA, respectively. The TEC of VMs is shown in Figure 5, which shows that the DAMPA consumes less energy. To obtain more accurate results, Table 8 lists the percentage of TEC optimization, the TEC is reduced by 6.30%, 27.04%, 38.60%, and 47.50% on average over IMMPA, HHO, MRFOSSA, and WOA, respectively. Figure 6 shows the system throughput for different VMs. The DAMPA achieves a greater throughput of all algorithms.

Summarizing case 1 and case 2, when there are many tasks with limited resources, the improvement of DAMPA based on other algorithms is up to 33.14% at most, when the available resources increase, DAMPA can reduce the makespan by 50.60% and the energy consumption by 47.50% at most.

## 6. Conclusions

This study proposes a novel meta-heuristic algorithm DAMPA for solving TSCC. In DAMPA, the predator crowding degree ranking strategy and comprehensive learning strategy are taken to maintain the diversity of the population, thereby avoiding premature convergence. In the second stage of DAMPA, the predators are selected to perform exploration or exploitation according to the ranking of the crowding degree of the predators, and a comprehensive learning strategy makes predators share the optimal historical experience. Additionally, to balance the exploitation and exploration capabilities of the algorithm, a stage-independent control of the stepsize-scaling strategy is designed, which uses different control parameters for scaling the step sizes in three stages. Two case experimental results show that DAMPA achieves lower makespan and energy consumption and greater throughput compared with the latest algorithms: IMMPA, HHO, MRFOSSA, and WOA. Especially in the second case, DAMPA has a distinct advantage in solving TSCC problems.

In the future, we will focus on applying the DAMPA algorithm to other problems, such as cloud-fog collaborative TS with constraints and cloud-edge collaborative TS.

## Figures and Tables

**Table 1:** Parameter setting.

Algorithm	Parameter	Value
DAMPA	P1	0.05
	P2	0.5
	P3	1
IMMPA	P	1
HHO	β	1.5
WOA	b	1
MRFOSSA	S	2

**Table 2:** Comparison based on fitness under different number of tasks.

Tasks	DAMPA	IMMPA	HHO	MRFOSSA	WOA
100	**1191**	1233	1426	1553	1800
200	**2603**	2730	2854	3167	3453
300	**3790**	3898	4065	4588	4491
400	**4989**	5081	5260	5939	5705
500	**6207**	6251	6614	7255	7167
600	**7490**	7515	7943	8496	8361
700	**8557**	8665	8725	9693	9093
800	**9710**	9781	10,036	10,908	10,676

**Table 3:** Comparison based on makespan under different number of tasks.

Tasks	DAMPA	IMMPA	HHO	MRFOSSA	WOA
100	**8.16**	8.37	9.22	9.74	11.89
200	**15.19**	16.40	16.87	19.24	20.82
300	**22.00**	23.10	24.08	26.72	26.69
400	**28.52**	29.14	29.83	33.70	32.55
500	**35.21**	35.79	37.44	40.28	39.73
600	**41.77**	41.80	44.25	48.53	48.17
700	**47.32**	47.93	48.22	55.33	51.20
800	**53.43**	53.43	55.70	62.34	62.05

**Table 4:** The improvement percentage in makespan under different number of tasks.

Algorithm	100	200	300	400	500	600	700	800
IMMPA	2.60%	7.38%	4.76%	2.14%	1.62%	0.07%	1.28%	0%
MRFOSSA	16.27%	21.06%	17.69%	15.38%	12.59%	13.93%	14.48%	14.30%
HHO	11.56%	9.99%	8.64%	4.41%	5.96%	5.60%	1.86%	4.08%
WOA	31.37%	27.05%	17.59%	12.40%	11.37%	13.28%	7.58%	13.89%

**Table 5:** Comparison based on TEC under different number of tasks.

Tasks	DAMPA	IMMPA	HHO	MRFOSSA	WOA
100	**2400**	2424	2915	3137	3591
200	**5108**	5453	5754	6356	6834
300	**7625**	8037	8321	9202	8954
400	**10,122**	10,294	10,382	11,922	11,378
500	**12,484**	12,741	13,236	14,433	14,280
600	**14,934**	14,946	15,753	16,993	16,674
700	**17,165**	17,245	17,490	19,413	18,185
800	**19,286**	19,719	19,989	21,859	21,316

**Table 6:** The improvement percentage in TEC under different number of tasks.

Algorithm	100	200	300	400	500	600	700	800
IMMPA	0.99%	6.33%	5.12%	1.67%	2.01%	0.07%	0.46%	0.11%
MRFOSSA	23.47%	19.63%	17.13%	15.10%	13.50%	12.11%	11.57%	11.76%
HHO	17.64%	11.22%	8.35%	2.50%	5.68%	5.19%	1.85%	3.51%
WOA	33.14%	25.25%	14.83%	11.04%	12.57%	10.43%	5.60%	9.52%

**Table 7:** The improvement percentage in makespan under different numbers of VMs.

Algorithm	50	60	70	80	90	100	110	120	130	Ave
IMMPA	10.48%	5.53%	7.07%	4.00%	4.64%	6.09%	6.33%	8.50%	8.68%	6.81%
MRFOSSA	41.76%	35.60%	36.11%	36.33%	31.64%	33.12%	33.00%	34.33%	27.27%	34.35%
HHO	30.43%	28.57%	26.53%	24.79%	19.27%	24.79%	28.89%	31.38%	27.63%	26.92%
WOA	48.77%	50.94%	54.63%	55.74%	43.26%	46.33%	51.12%	48.65%	55.94%	50.60%

**Table 8:** The improvement percentage in TEC under different numbers of VMs.

Algorithm	50	60	70	80	90	100	110	120	130	Ave
IMMPA	6.97%	2.40%	8.39%	11.11%	3.77%	6.10%	9.08%	4.15%	4.69%	6.30%
MRFOSSA	40.32%	36.14%	40.76%	43.65%	36.02%	37.61%	40.16%	37.41%	35.37%	38.60%
HHO	30.82%	23.23%	31.51%	30.60%	21.41%	27.46%	29.33%	25.65%	23.34%	27.04%
WOA	48.46%	33.39%	54.38%	49.19%	41.11%	49.75%	53.25%	44.35%	53.61%	47.50%

## Data Availability

Not applicable.

## References

[1] Rimal B.P., Maier M. (2017). Workflow Scheduling in Multi-Tenant Cloud Computing Environments. IEEE Trans. Parallel Distrib. Syst..

[2] Abualigah L., Diabat A., Sumari P., Gandomi A.H. (2021). Applications, Deployments, and Integration of Internet of Drones (IoD): A Review. IEEE Sens. J..

[3] Houssein E.H., Gad A.G., Wazery Y.M., Suganthan P.N. (2021). Task Scheduling in Cloud Computing Based on Meta-Heuristics: Review, Taxonomy, Open Challenges, and Future Trends. Swarm Evol. Comput..

[4] Yang Y., Shen H. (2021). Deep Reinforcement Learning Enhanced Greedy Algorithm for Online Scheduling of Batched Tasks in Cloud in Cloud HPC Systems. IEEE Trans. Parallel Distrib. Syst..

[5] Velliangiri S., Karthikeyan P., Arul Xavier V.M., Baswaraj D. (2021). Hybrid Electro Search with Genetic Algorithm for Task Scheduling in Cloud Computing. Ain Shams Eng. J..

[6] Shukla D.K., Kumar D., Kushwaha D.S. (2021). WITHDRAWN: Task Scheduling to Reduce Energy Consumption and Makespan of Cloud Computing Using NSGA-II. Mater. Today Proc..

[7] Cui Z., Zhang J., Wu D., Cai X., Wang H., Zhang W., Chen J. (2020). Hybrid Many-Objective Particle Swarm Optimization Algorithm for Green Coal Production Problem. Inf. Sci..

[8] Abd Elaziz M., Attiya I. (2021). An Improved Henry Gas Solubility Optimization Algorithm for Task Scheduling in Cloud Computing. Artif. Intell. Rev..

[9] Heidari A.A., Mirjalili S., Faris H., Aljarah I., Mafarja M., Chen H. (2019). Harris Hawks Optimization: Algorithm and Applications. Future Gener. Comput. Syst..

[10] Faramarzi A., Heidarinejad M., Mirjalili S., Gandomi A.H. (2020). Marine Predators Algorithm: A Nature-Inspired Metaheuristic. Expert Syst. Appl..

[11] Marahatta A., Pirbhulal S., Zhang F., Parizi R.M., Choo K.-K.R., Liu Z. (2021). Classification-Based and Energy-Efficient Dynamic Task Scheduling Scheme for Virtualized Cloud Data Center. IEEE Trans. Cloud Comput..

[12] Hussain M., Wei L.-F., Lakhan A., Wali S., Ali S., Hussain A. (2021). Energy and Performance-Efficient Task Scheduling in Heterogeneous Virtualized Cloud Computing. Sustain. Comput. Inform. Syst..

[13] Chen X., Cheng L., Liu C., Liu Q., Liu J., Mao Y., Murphy J. (2020). A WOA-Based Optimization Approach for Task Scheduling in Cloud Computing Systems. IEEE Syst. J..

[14] Abdullah M., Al-Muta’a E.A., Al-Sanabani M. (2019). Integrated MOPSO Algorithms for Task Scheduling in Cloud Computing. IFS.

[15] Laili Y., Guo F., Ren L., Li X., Li Y., Zhang L. (2021). Parallel Scheduling of Large-Scale Tasks for Industrial Cloud-Edge Collaboration. IEEE Internet Things J..

[16] Ali I.M., Sallam K.M., Moustafa N., Chakraborty R., Ryan M.J., Choo K.-K.R. (2020). An Automated Task Scheduling Model Using Non-Dominated Sorting Genetic Algorithm II for Fog-Cloud Systems. IEEE Trans. Cloud Comput..

[17] Xiong Y., Huang S., Wu M., She J., Jiang K. (2019). A Johnson’s-Rule-Based Genetic Algorithm for Two-Stage-Task Scheduling Problem in Data-Centers of Cloud Computing. IEEE Trans. Cloud Comput..

[18] Pirozmand P., Hosseinabadi A.A.R., Farrokhzad M., Sadeghilalimi M., Mirkamali S., Slowik A. (2021). Multi-Objective Hybrid Genetic Algorithm for Task Scheduling Problem in Cloud Computing. Neural Comput. Appl..

[19] Pang S., Li W., He H., Shan Z., Wang X. (2019). An EDA-GA Hybrid Algorithm for Multi-Objective Task Scheduling in Cloud Computing. IEEE Access.

[20] Xu J., Hao Z., Zhang R., Sun X. (2019). A Method Based on the Combination of Laxity and Ant Colony System for Cloud-Fog Task Scheduling. IEEE Access.

[21] Attiya I., Elaziz M.A., Abualigah L., Nguyen T.N., El-Latif A.A.A. (2022). An Improved Hybrid Swarm Intelligence for Scheduling IoT Application Tasks in the Cloud. IEEE Trans. Ind. Inf..

[22] Walia N.K., Kaur N., Alowaidi M., Bhatia K.S., Mishra S., Sharma N.K., Sharma S.K., Kaur H. (2021). An Energy-Efficient Hybrid Scheduling Algorithm for Task Scheduling in the Cloud Computing Environments. IEEE Access.

[23] Domanal S.G., Guddeti R.M.R., Buyya R. (2020). A Hybrid Bio-Inspired Algorithm for Scheduling and Resource Management in Cloud Environment. IEEE Trans. Serv. Comput..

[24] Fu X., Sun Y., Wang H., Li H. (2021). Task Scheduling of Cloud Computing Based on Hybrid Particle Swarm Algorithm and Genetic Algorithm. Clust. Comput..

[25] Abdel-Basset M., El-Shahat D., Elhoseny M., Song H. (2021). Energy-Aware Metaheuristic Algorithm for Industrial-Internet-of-Things Task Scheduling Problems in Fog Computing Applications. IEEE Internet Things J..

[26] Yousri D., Fathy A., Rezk H. (2021). A New Comprehensive Learning Marine Predator Algorithm for Extracting the Optimal Parameters of Supercapacitor Model. J. Energy Storage.

[27] Abdel-Basset M., Mohamed R., Elhoseny M., Bashir A.K., Jolfaei A., Kumar N. (2021). Energy-Aware Marine Predators Algorithm for Task Scheduling in IoT-Based Fog Computing Applications. IEEE Trans. Ind. Inf..

